# Impacts of bioturbation on temporal variation in bacterial and archaeal nitrogen-cycling gene abundance in coastal sediments

**DOI:** 10.1111/1758-2229.12115

**Published:** 2013-11-19

**Authors:** B Laverock, K Tait, J A Gilbert, A M Osborn, S Widdicombe

**Affiliations:** 1 Plymouth Marine LaboratoryProspect Place, Plymouth, PL1 3DH, UK; 2 Department of Animal and Plant Sciences, University of SheffieldSheffield, S10 2TN, UK; 3 School of Plant Biology and the UWA Oceans Institute, University of Western AustraliaCrawley, WA, 6009, Australia; 4 Argonne National Laboratory, Institute of Genomic and Systems Biology9700 South Cass Avenue, Argonne, IL, 60439, USA; 5 Department of Ecology and Evolution, University of Chicago5640 South Ellis Avenue, Chicago, IL, 60637, USA; 6 Department of Biological Sciences, University of HullHull, HU6 7RX, UK; † School of Life Sciences, University of Lincoln, Brayford PoolLincoln, LN6 7TS, UK

## Abstract

In marine environments, macrofauna living in or on the sediment surface may alter the structure, diversity and function of benthic microbial communities. In particular, microbial nitrogen (N)-cycling processes may be enhanced by the activity of large bioturbating organisms. Here, we study the effect of the burrowing mud shrimp *Upogebia deltaura* upon temporal variation in the abundance of genes representing key N-cycling functional guilds. The abundance of bacterial genes representing different N-cycling guilds displayed different temporal patterns in burrow sediments in comparison with surface sediments, suggesting that the burrow provides a unique environment where bacterial gene abundances are influenced directly by macrofaunal activity. In contrast, the abundances of archaeal ammonia oxidizers varied temporally but were not affected by bioturbation, indicating differential responses between bacterial and archaeal ammonia oxidizers to environmental physicochemical controls. This study highlights the importance of bioturbation as a control over the temporal variation in nitrogen-cycling microbial community dynamics within coastal sediments.

## Introduction

Bioturbation – the physical and chemical disturbance of a sediment body by macrofauna or meiofauna – can impact upon microbial community dynamics within sediments. For example, the presence of thalassinidean shrimp species *Upogebia deltaura* and *Callianassa subterranea*, which are active and abundant decapods that create large, complex burrow systems in marine sediments (Griffis and Suchanek, 1991) has been shown to significantly alter the structure and diversity of sediment bacterial communities (Laverock *et al*., 2010). Previous studies have indicated that the overall structure of microbial communities in shrimp burrow sediments may experience different seasonal patterns from those in surface sediments. Within burrows of another thalassinidean shrimp (*Pestarella tyrrhena*), bacterial communities exhibited less seasonal change in structure than those communities inhabiting surface sediment (Papaspyrou *et al*., 2005). Conversely, bacterial communities within *U. major* burrows were more seasonally changeable (measured as both cell abundance and electron transport activity) compared with those within tidal flat surface and subsurface sediments (Kinoshita *et al*., 2008).

Shrimp burrows have also previously been associated with enhanced nitrification potential in the burrow walls, increased rates of denitrification in surrounding bulk sediment and increased efflux of dissolved inorganic nitrogen from the sediment (DeWitt *et al*., 2004; Howe *et al*., 2004; Webb and Eyre, 2004). Any temporal variation in the effects of bioturbation on the abundance and activity of nitrogen-cycling microorganisms could therefore alter the seasonal transformations and fluxes of nitrogen across the sediment-water interface.

Here, we use the quantitative polymerase chain reaction (q-PCR) to investigate temporal variation in the abundance of specific bacterial and archaeal genes representing key N-cycling functional guilds within sediment samples taken from the walls of *U. deltaura* burrows in comparison with their abundances within ambient surface sediment samples.

## Results and discussion

### Shrimp burrows alter the temporal variability of specific microbial genes

The q-PCR was used to enumerate bacterial and archaeal 16S rRNA genes, as well as genes representing betaproteobacterial and archaeal ammonia oxidizers (*amoA*); bacterial denitrifiers (*nirS*) and bacteria capable of the anammox process (*Planctomycetes*-specific 16S rRNA). Clone library analysis confirmed the specificity of each of the primer pairs used for subsequent q-PCR. In particular, sequence analysis showed that the *Planctomycetes*-specific 16S rRNA primers used here specifically targeted anammox bacteria, as shown previously (Jayakumar *et al*., 2009; Hu *et al*., 2011; Sonthiphand and Neufeld, 2013); these samples are henceforth labelled as anammox 16S rRNA.

**Figure 1:**
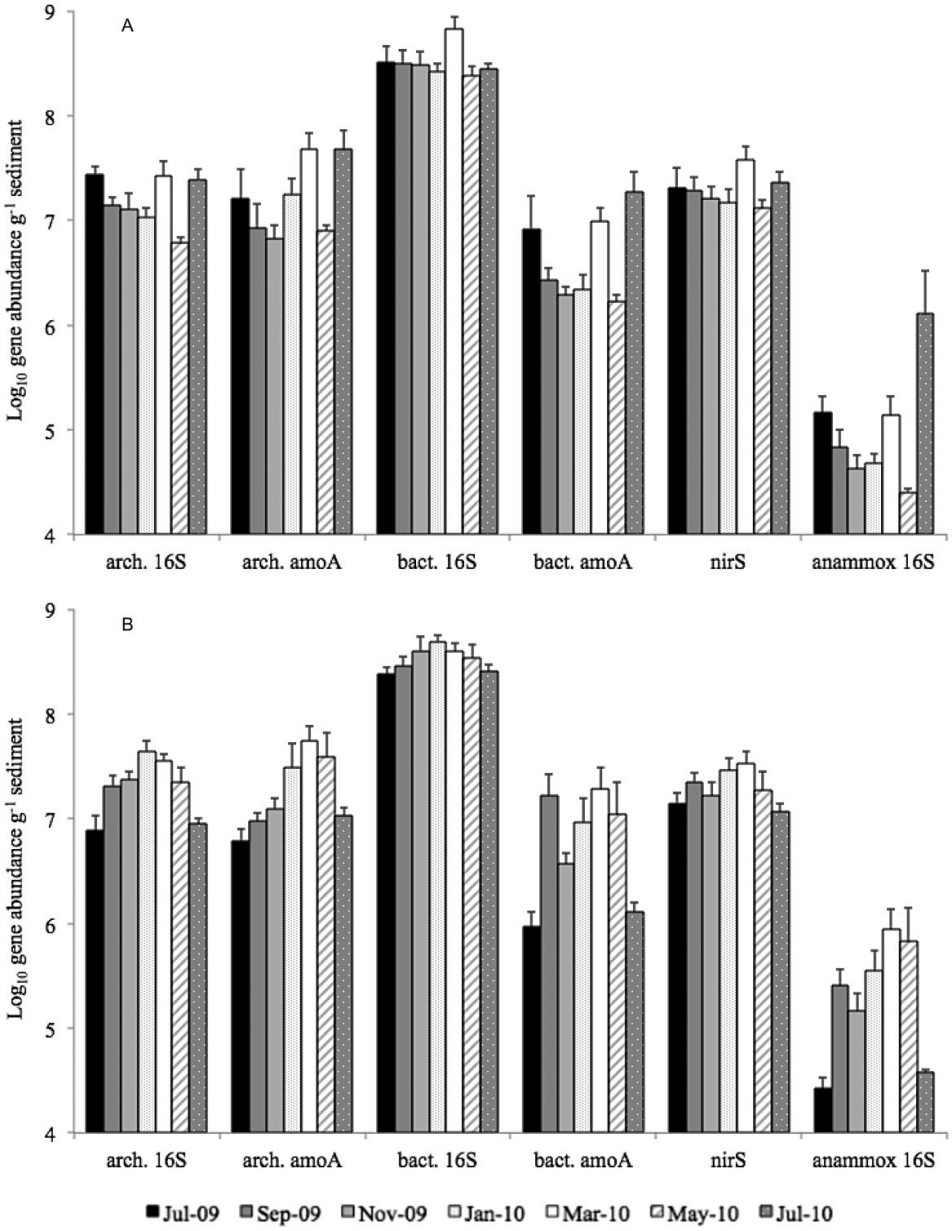
Seasonal variation in the abundance of bacterial and archaeal 16S rRNA and nitrogen-cycling functional genes in (A) surface and (B) burrow sediments. In order to compare abundance patterns across all genes, log_10_ abundances are plotted. Error bars show standard error based on five replicates for sample, except for surface samples from Jul-09 and burrow samples from Jul-10, where *n* = 4. Gene abundances were calculated from standard curves using the *r*^2^, *y* intercept and efficiency values given in Supporting Information Table S2; gene abundance data are also shown in Table S3.

Variation in gene abundances was considered with respect to the *a priori* group factors ‘sediment category’ (surface vs. burrow sediment) and ‘sampling month’. Bacterial 16S rRNA and *nirS* gene abundances showed no significant variation with either sediment category or sampling month. However, the abundances of each of the other genes studied (archaeal 16S rRNA, archaeal and bacterial *amoA*, and anammox 16S rRNA) varied significantly with sampling month (Fig. 1; Table 1a). This temporal variation was often greater when sediment category and sampling month were considered as crossed factors (Table 1a), indicating that there were different temporal patterns in gene abundances occurring between surface and burrow sediments. Significant differences in abundance between sediment categories was only found for anammox 16S rRNA genes, which were on average 6.8 times more abundant within burrow sediments than within surface sediments (*F* 7.80, *P* < 0.01; Table 2). However, for all genes, the ratio of genes in surface sediments compared with those in burrow sediments was temporally variable; with in general, surface sediments only containing higher gene abundances (ratios of < 1) during the summer months (July 2009 and July 2010) (Table 2).

**Table 1:** Multivariate analyses of microbial gene abundance over a 1-year period in surface and bioturbated (burrow wall) sediment

	Sediment	Month	Se × Mo
Gene abundance			
Archaeal 16S rRNA	3.36	3.51*	9.33*
Archaeal *amoA*	0.93	6.52*	2.29*
Bacterial 16S rRNA	0.24	1.83	1.17
Bacterial *amoA*	0.10	3.89*	7.03*
*nirS*	0.03	2.06	1.01
Anammox 16S rRNA	7.80*	**2.56**	9.00*
Relative abundance of *amoA, nirS* and anammox 16S rRNA genes			
Archaeal *amoA*	0.87	6.32*	2.16
Bacterial *amoA*	0.91	3.31*	8.93*
*nirS*	0.25	**2.46**	3.11*
Anammox 16S rRNA	13.18*	**2.49**	12.73*

Significant variation was explored between sediment category (surface vs. burrow) and sampling month, and sediment category crossed with sampling month (Se × Mo), for (a) gene abundances, and (b) relative abundance of *amoA, nirS* and anammox 16S rRNA genes in comparison with archaeal and bacterial 16S rRNA genes. PERMANOVA tests were performed in PRIMER 6.1 (Clarke and Gorley, 2006) using the PERMANOVA+ add-on (beta version, Anderson *et al*., 2008), on Bray–Curtis resemblance matrices calculated from log(x + 1)-transformed abundance data. *F* values significant at *P* < 0.05 are highlighted in bold; those values significant at *P* < 0.01 are also indicated (*). Data for absolute gene abundances are summarized in the Supporting Information (Table S3).

**Table 2:** Seasonal variation in the mean ratios of genes in burrow: surface sediments

	Archaeal 16S rRNA	Archaeal *amoA*	Bacterial 16S rRNA	Bacterial *amoA*	*nirS*	Anammox 16S rRNA
Jul-09	0.28	0.39	0.74	0.11	0.70	0.18
Sep-09	1.49	1.13	0.93	6.16	1.16	3.77
Nov-09	1.85	1.89	1.34	1.90	1.03	3.47
Jan-10	4.11	1.74	1.86	4.24	1.98	7.34
Mar-10	1.35	1.15	0.58	1.95	0.89	6.41
May-10	3.59	4.92	1.41	6.56	1.43	26.50
Jul-10	0.36	0.18	0.74	0.06	0.41	0.02
Mean	1.86	1.63	1.08	3.00	1.09	6.81

**Figure 2:**
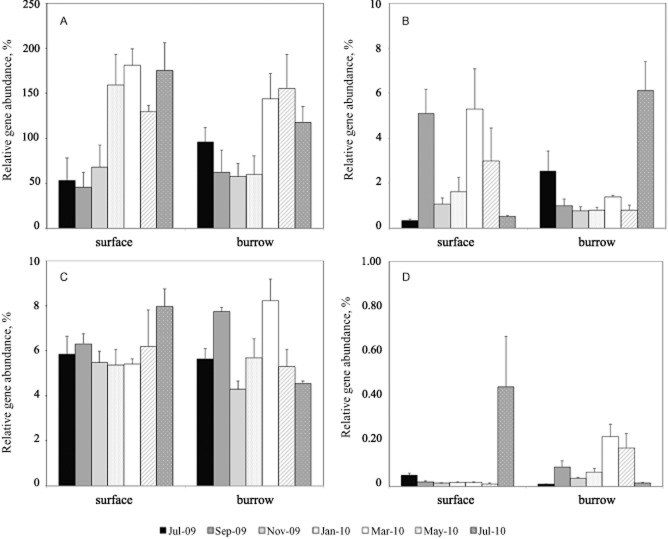
Relative abundance of genes representing bacterial and archaeal functional guilds in surface and burrow sediments: (A) archaeal *amoA*, (B) bacterial *amoA*, (C) *nirS* and (D) anammox 16S rRNA genes. The *y* axis represents the % abundance of each functional gene, normalized to the appropriate (bacterial or archaeal) 16S rRNA gene abundance; scale differs for each plot. Error bars show standard error based on five replicates for sample, except for surface samples from Jul-09 and burrow samples from Jul-10, where *n* = 4.

In order to investigate potential changes in gene abundances representing different N-cycling guilds in relation to the changes in abundance of the overall microbial communities, we determined the relative abundance of each functional gene as a percentage of the total number of 16S rRNA genes (bacterial or archaeal, as relevant) (Fig. 2). The method of comparing functional gene abundance with 16S rRNA abundance is used here with caution, given that varying copy numbers of each gene per cell prevents us from discussing the absolute proportion of functional genes within the total community. For example, it is assumed that the betaproteobacterial *amoA* gene is present in two or three copies per cell (Arp *et al*., 2007), whereas evidence for archaeal ammonia oxidizers suggests only a single *amoA* copy per genome (Walker *et al*., 2010; Blainey *et al*., 2011). Meanwhile, denitrifying bacteria and archaea may possess between one and three copies of the *nirS* gene (Zumft, 1997; Jones *et al*., 2008). Moreover, it is known that between 1 and 14 copies of bacterial 16S rRNA can exist in each cell (Farrelly *et al*., 1995), whereas the evidence so far indicates that marine *Crenarchaeaota*, to which phylum the archaeal ammonia oxidizers belong, contain only one rRNA operon per genome (Klappenbach *et al*., 2001; Mincer *et al*., 2007).

Comparing functional gene relative abundance data with the absolute abundances shown in Fig. 1, some new patterns were revealed. In particular, the relative abundance of *nirS* varied significantly with sampling month, and as for bacterial *amoA* and anammox 16S rRNA, there was a significant interaction between sampling month and sediment category (Table 1b). In contrast, for the archaeal *amoA* gene, significant variation in relative abundance occurred temporally, but a combined sediment category X temporal effect was no longer observed (Fig. 2; Table 1).

### Differential controls on N-cycling gene abundance in shrimp burrows compared with surface sediments

In general, the abundances of all genes in surface sediments were highest in both July 2009 and 2010, and lowest in May, with an additional peak in March (Fig. 1). In contrast, gene abundances in burrow sediments followed a hump-shaped pattern between July 2009 and July 2010, with abundances peaking in January or March (Fig. 1). The RELATE test performed using the PRIMER 6.1 software for multivariate analysis (Clarke and Gorley, 2006) showed significant (seasonal) cyclicity to the patterns in microbial gene abundances within burrow sediments (*ρ* = 0.405, *P* < 0.05); this effect was not evident for surface sediments. We may interpret these differing seasonal patterns in gene abundance within the context of environmental factors known to impact upon microbial community dynamics in coastal sediments.

For example, variation in microbial community abundance and activity in surface sediments is generally assumed to be driven by changing environmental conditions, such as pH and salinity, or stochastic processes, such as sediment turnover during a storm. On seasonal timescales, the most important regulators of N-cycling functional guild abundance may be temperature, which exerts a major control over all life processes (e.g. Nedwell and Rutter, 1991; Pomeroy and Wiebe, 2001), and substrate availability in the form of organic matter or inorganic nutrients. For our study site of Jennycliff Bay, as for other coastal sediments, inorganic nutrient concentrations in the overlying water generally increase during the winter, when terrestrial run-off is high (Fig. S1). This readily available source of nutrients is accompanied by an increase in water temperature in spring, allowing enhanced microbial growth and productivity (Kristensen, 1993), and hence, an increase in gene abundances such as those seen here for surface sediments in March (Fig. 1).

The sharp decline in nutrient concentrations between March and May is concomitant with the occurrence of the phytoplankton spring bloom in the Western English Channel (Smyth *et al*., 2010). Increasing autotrophic activity (both pelagic and benthic) during spring may affect the abundance of benthic microbial N-cycling genes in two ways. First, greater competition for nutrients (during the day) and O_2_ (during the night) with the blooming populations of autotrophs has been shown to adversely affect benthic ammonia oxidizing bacteria (AOB) abundance (Risgaard-Petersen, 2003; Risgaard-Petersen *et al*., 2004). Presumably, this effect may extend to other N-cycling genes. Second, several previous studies have shown that the deposition of phytodetritus during and after spring bloom periods can significantly alter microbial community structure and activity in coastal sediments (e.g. Graf *et al*., 1982; 1983; Meyer-Reil, 1983).

In contrast with the primarily environment-driven processes in surface sediments, the shrimp burrow is considered to be a unique environment, in which physicochemical conditions are largely controlled by shrimp behaviour and to some extent decoupled from the pelagic realm. We have observed a lower abundance of N-cycling genes in shrimp burrow sediments in summer than in winter (Fig. 1), which may be related to increased shrimp activities during summer. For example, burrow irrigation events – during which the shrimp beat their pleopods (walking legs) to flush oxygenated water through the burrow – exert a major control on the redox potential of burrow walls, causing oscillations in pH, as well as in the partial pressure of oxygen (*p*O_2_), during irrigation events (Astall *et al*., 1997; Stamhuis and Videler, 1998). Both irrigation activity and burrow maintenance behaviour, which causes physical disturbance of the burrow wall, have been shown to increase at higher temperatures (Berkenbusch and Rowden, 1999; Stanzel and Finelli, 2004), corresponding with the enhanced availability of labile organic matter within the burrow wall during the summer months (Kristensen, 2000; Papaspyrou *et al*., 2005; Kinoshita *et al*., 2008). It is therefore reasonable to expect that the nitrogen-cycling microbial communities inhabiting burrow walls are regulated by the higher level of physicochemical disturbance imposed upon them by shrimp behaviour in summer months, potentially explaining the lower gene abundances observed here. During the winter months, lower levels of bioturbation activity may act in combination with the greater availability of nutrients typical of temperate coastal zones (Canfield *et al*., 2005) to encourage microbial assemblages to flourish within the burrow environment. How this may relate to the seasonally variable rates of nitrogen cycling processes in these sediments remains to be seen.

### High abundance and temporal variability of archaeal *amoA* genes

Although bacterial 16S rRNA genes were, on average, 17 times more abundant than archaeal 16S rRNA genes, archaeal *amoA* genes were four times more abundant than betaproteobacterial *amoA* genes throughout the year and in both sediment categories, except in burrow sediments in September 2009 (data not shown). Ammonia oxidizing archaea (AOA) have been found in greater abundance than AOB in a variety of benthic marine and estuarine environments (e.g. Caffrey *et al*., 2007; Mosier and Francis, 2008; Santoro *et al*., 2008; Moin *et al*., 2009; Abell *et al*., 2010; Bernhard *et al*., 2010). We have shown here that the relative abundance of AOB in sediment is significantly affected by shrimp bioturbation, whereas AOA relative abundance is seemingly subject to temporally variable controls that are independent of bioturbation (Fig. 2). Winter bloom dynamics, characterized by dramatic increases in both 16S rRNA and *amoA* abundance during winter, have previously been reported for AOA in the water column (e.g. Herfort *et al*., 2007; Pitcher *et al*., 2011). One reason for this may be the increased availability of ammonium during winter months (Pitcher *et al*., 2011); however, other factors have been shown to influence the abundance of AOA in various environments. For example, AOA abundances have been linked to salinity gradients in sediment (Caffrey *et al*., 2007; Santoro *et al*., 2008; Bernhard *et al*., 2010) and dissolved oxygen levels in the water column (Lam *et al*., 2007).

It should be noted that the relative abundance of archaeal *amoA* genes was often greater than 100%, apparently indicating a higher abundance of *amoA* than of 16S rRNA genes (Fig. 2). Previously, the archaeal *amoA*: 16S rRNA ratio has been used to suggest multiple (≥ 2) *amoA* copies per cell (Wuchter *et al*., 2006), as well as to infer different ‘ecotypes’ corresponding to the redox state of the water column in the Black Sea (Lam *et al*., 2007). However, considering the genomic evidence thus far suggests that AOA contain only one copy of the *amoA* gene per genome (e.g. Walker *et al*., 2010; Blainey *et al*., 2011), it may be more likely that discrepancies between 16S rRNA and *amoA* gene abundances can be attributed to unspecificity or bias in one of both of the primer sets used here. Nevertheless, we have shown that the dominance of the *amoA* gene within the total archaeal community was distinctly seasonal (*F* 6.32, *P* < 0.01; Fig. 2), potentially indicating a community shift during summer months. Recently, Sintes and colleagues (2013) have identified two ecotypes for AOA in the water column, corresponding to high-ammonia and low-ammonia regions. The temporal variability of AOA relative abundance observed in this current study may therefore be related to seasonally variable environmental conditions, such as O_2_ or NH_3_ availability, or directly to temperature variation.

It remains uncertain whether AOB or AOA make a greater contribution to ammonia oxidation rates in marine systems, or indeed, whether all AOA are obligate ammonia oxidizers (Santoro *et al*., 2010; Muβmann *et al*., 2011; Hatzenpichler, 2012; Stahl and de la Torre, 2012). However, we have shown that it is likely that the presence of bioturbating shrimp has a significant impact upon the temporal abundances of these microbial groups, indicating the ecological importance of the relationship between macrofaunal and microbial sediment inhabitants. The relative activity and importance of AOB and AOA in the global nitrogen cycle is therefore likely to be strongly dependent on both habitat type and season.

### *N_2_* loss may be enhanced by bioturbation

The activity of *U. deltaura* enhances denitrification and coupled nitrification-denitrification by 2.9 and 3.3 times respectively (Howe *et al*., 2004). Here, we have also shown that bioturbation has a significant impact upon temporal patterns in the abundances of genes representing key bacterial guilds responsible for removal of reactive nitrogen N_r_ (e.g. NO_3_^−^, NO_2_^−^ and NH_4_^+^) from marine sediments by denitrification (*nirS*) and anammox. N_r_-removal processes play a critical role in coastal sediments, where terrestrial run-off can stimulate eutrophication events in coastal waters, particularly during autumn, when both precipitation rates and the input of agricultural nutrients into estuarine waters are high (Beman *et al*., 2005; Fig. S1). While the corresponding process rate measurements were unavailable for this current study, previous studies have suggested that bioturbation may reduce the effects of eutrophication by enhancing both organic matter burial and the rates of N_r_-removal processes (Tuominen *et al*., 1999; DeWitt *et al*., 2004). In Plymouth Sound denitrification, rather than anammox, is the dominant N_r_ removal process throughout the year, although both processes appear to vary proportionately with season, with lowest rates being observed in winter (V. Kitidis, unpubl. data). The impacts of bioturbation on the activity of the denitrifying bacteria could therefore be an important consideration in future models of the benthic response to coastal environmental change.

### Conclusions

Our data indicate that temporal variation in the abundance of N-cycling functional guilds (genes) is directly influenced by bioturbation activity, and such effects vary between different bacterial and archaeal N-cycling guilds. These data are interpreted with the caveat that the PCR primers used here target specific bacterial and archaeal phylotypes and/or ecotypes; for example, our primers for archaeal *amoA* target specifically the ‘high-ammonium’ ecotype identified by Sintes and colleagues (2013). We also report the abundances only of *nirS*-type denitrification genes and betaproteobacterial *amoA* genes. It has been previously reported that gammaproteobacterial ammonia oxidizers may have a limited diversity but widespread occurrence in marine sediments and oxygen minimum zones (Ward and O’Mullan, 2002; Lam *et al*., 2007), while *nirK*-type denitrification genes have been found to be less diverse but equal in abundance to *nirS* within Black Sea water samples (Jayakumar *et al*., 2009). Both of these genes failed to amplify within our samples; presumably either because of low copy number, inhibition by humic substances or because the primer sets used did not provide suitable coverage of the genes present within this specific environment (Braker *et al*., 2000; Throbäck *et al*., 2004). In addition, *nirK* and *nirS* gene homologues are widespread within the archaea, including AOA (e.g. Philippot, 2002; Cabello *et al*., 2004; Bartossek *et al*., 2010; Hatzenpichler, 2012). The PCR primers used in this current study were specific to bacterial *nirS* (Braker *et al*., 2000; Throbäck *et al*., 2004), and no molecular probes have yet been designed that target archaeal *nirS* (Rusch, 2013). However, clearly, the contribution of the AOA to denitrification represents an understudied pathway within the nitrogen cycle. Finally, the proportion of anammox bacteria may be significantly underestimated by amplification of the *Planctomycetes-* or anammox-specific 16S rRNA gene (Li *et al*., 2010). It is becoming more common, therefore, to alternatively investigate abundances of anammox bacteria using primers targeting the *hzo* gene, which encodes the hydrazine oxidoreductase protein, a key component of the anammox process (e.g. Schmid *et al*., 2008; Li *et al*., 2010).

It is also worth noting that the presence of a functional gene does not mean that function is operating within the specific environment, and an important continuation to this study would be to identify whether these genes correlate with the relevant biogeochemical rate processes. Nevertheless, we have observed gene abundances that fit within the ranges of previously reported values for these genes in similar marine environments (Table S3) and vary significantly with the *a priori* factors used here: sediment category (surface vs. burrow) and sampling month. This study therefore highlights the importance of temporal variation coupled to macrofaunal bioturbation activity in driving microbial community dynamics in coastal sediments. In particular, this study emphasizes the importance of the often overlooked interactions between macrofaunal and microbial communities upon key biogeochemical cycling processes within coastal benthic ecosystems. Our suggestions for the ways in which shrimp behaviour influence gene abundance patterns may act as a useful starting point for future investigations into the environmental controls on microbial functional diversity and activity.

## Supporting Information

**Fig. S1.** Variation in monthly pelagic nutrient concentrations in Jennycliff Bay (5.3497 N, 04.1331 W) in the Western English Channel. (A) nitrite, (B) nitrate, (C) ammonium, (D) silicate, (E) phosphate. Inset legends show water depth in metres. Data are from the PML Benthic Survey Data Inventory (Woodward *et al*., 2013). (F) Variation in monthly temperature (closed circles; black line) and salinity (open diamonds; grey line) for the L4 site (50.225 N, 04.1944 W), in the Western English Channel, with lines connecting the average value for each month. Data are from the Western Channel Observatory Data Inventory (Fishwick, 2013). **Table S1.** Sediment sampling strategy. **Table S2.** Primer pairs and reaction conditions used for quantitative polymerase chain reaction (q-PCR) assays. q-PCR amplification and detection for all assays was carried out using an ABI 7000 sequence detection system (Applied Biosystems, Carlsbad CA, USA) with an initial denaturation for 5 min at 95°C, followed by 40 cycles of 95°C for 15 s and annealing temperatures as listed below for 1 min. All reactions were carried out in 25 μl final volume; final primer concentrations are shown for this reaction volume. For each reaction, the standard curve was calculated using the ABI Prism 7000 sequence detection software. From the standard curve, the slope (*m*), *y* intercept and coefficient of determination (*r*^2^) recorded and used to calculate the efficiency of the amplification (E) using the equation E = (10^1/^*^m^* − 1) × 100. Values for calculating the efficiency of each reaction are given, as well as the threshold cycle value (*C_T_*), which was determined using automatic analysis settings. **Table S3.** Seasonal variation in the average gene abundance (gene copies g^−1^ wet sediment) in burrow (B) and surface (S) sediments, and comparison to abundances reported in the literature for similar marine environments and using similar gene primers. Full data are available from the Dryad Digital Repository (doi: to be confirmed). Absolute gene abundances were calculated from standard curves using the *r*^2^, *y* intercept and efficiency values given in Table S2.
