# Supplementary material for: Impacts of bioturbation on temporal variation in bacterial and archaeal nitrogen-cycling gene abundance in coastal sediments
Source: Environ Microbiol Rep. 2013 Nov 19;6(1):113–21. doi: 10.1111/1758-2229.12115 (PMC4208606; doi:10.1111/1758-2229.12115)
Supplement: Supplementary file 1 — Fig. S1. Variation in monthly pelagic nutrient concentrations in Jennycliff Bay (5.3497 N, 04.1331 W) in the Western English Channel. (A) nitrite, (B) nitrate, (C) ammonium, (D) silicate, (E) phosphate. Inset legends show water depth in metres. Data are from the PML Benthic Survey Data Inventory (Woodward et al., 2013). (F) Variation in monthly temperature (closed circles; black line) and salinity (open diamonds; grey line) for the L4 site (50.225 N, 04.1944 W), in the Western English Channel, with lines connecting the average value for each month. Data are from the Western Channel Observatory Data Inventory (Fishwick, 2013). Table S1. Sediment sampling strategy. Table S2. Primer pairs and reaction conditions used for quantitative polymerase chain reaction (q-PCR) assays. q-PCR amplification and detection for all assays was carried out using an ABI 7000 sequence detection system (Applied Biosystems, Carlsbad CA, USA) with an initial denaturation for 5 min at 95°C, followed by 40 cycles of 95°C for 15 s and annealing temperatures as listed below for 1 min. All reactions were carried out in 25 μl final volume; final primer concentrations are shown for this reaction volume. For each reaction, the standard curve was calculated using the ABI Prism 7000 sequence detection software. From the standard curve, the slope (m), y intercept and coefficient of determination (r2) recorded and used to calculate the efficiency of the amplification (E) using the equation E = (101/m − 1) × 100. Values for calculating the efficiency of each reaction are given, as well as the threshold cycle value (CT), which was determined using automatic analysis settings. Table S3. Seasonal variation in the average gene abundance (gene copies g−1 wet sediment) in burrow (B) and surface (S) sediments, and comparison to abundances reported in the literature for similar marine environments and using similar gene primers. Full data are available from the Dryad Digital Repository (doi: to be confirmed). Ab [file emi40006-0113-sd1.docx]

**Impacts of bioturbation on temporal variation in bacterial and archaeal nitrogen-cycling gene abundance in coastal sediments**

B. Laverock, K. Tait, J.A. Gilbert, A.M. Osborn, S. Widdicombe

**Electronic Supplementary Materials**

*Sediment sampling strategy*

Between July 2009 and July 2010, sediment was collected every two months from burrow and surface sediments from a site in Jennycliff Bay, Plymouth Sound (50.346 N, 04.127 W; 10 m water depth). Sediment was collected using a 0.1 m^2^ box corer and placed on deck in order to collect samples for molecular analysis (Table S1).

Surface sediment samples were taken immediately, and then the core was gently broken apart by hand to search for any burrows present. For both surface and burrow sediments, samples of approximately 1.5 ml volume were transferred into 2 ml Eppendorf tubes using a sterile steel spatula, and immediately frozen in liquid nitrogen, prior to storage at -80 °C. “Surface” sediment was scraped from the top ~5 mm oxidised layer indicated by a lighter sediment colour. “Burrow” sediment was scraped from the oxidised layer (~3 mm) representing the burrow wall; care was taken to collect sediment only from the burrows of *U. deltaura* (identified visually), and sediment was scraped from the “mid-burrow” section (as defined by Laverock *et al*., 2010) at depths greater than 3 cm, but not representing the burrow “sump”.

*Molecular analysis using q-PCR*

DNA was extracted using a bead-beating and phenol-chloroform technique, as previously described (Laverock *et al*., 2010), and purified using the MoBio PowerClean DNA Clean-up Kit (Cambio, Cambridge, UK). Numbers of bacterial and archaeal 16S rRNA, bacterial and archaeal *amoA*, bacterial *nirS* genes, and *Planctomycetes*-specific 16S rRNA genes as a proxy for anammox bacteria, were determined using the quantitative polymerase chain reaction (q-PCR). Prior to q-PCR assays, the specificity of the primer set for each target gene was checked by cloning and sequencing, and the resulting PCR product was used to construct standard curves for the quantification of gene abundances. Briefly, target gene amplicons were then amplified from a subset of DNA samples, using the primer pairs and annealing temperatures listed in Table S2. PCR products were cloned using the pGEM-T Easy Vector System I cloning kit according to the manufacturer’s instructions (Promega, Southampton, UK), andtransformants were selected on Luria-Bertani agar plates containing ampicillin (50 mg ml^-1^), X-gal (40 mg ml^-1^) and 0.1 M IPTG. White colonies were screened by PCR using the vector primers M13F and M13R (Invitrogen, Paisley, UK) in a 50-μl PCR mixture containing 5X PCR buffer (Promega, Southampton, UK), 2 mM MgCl_2_, 0.2 mM dNTPs (dATP, dCTP, dGTP, dTTP) (Promega), 5 μg of BSA, 2.5 U of GoTaq Flexi DNA polymerase (Promega) and 1 µM of each primer. Reactions were initially denatured for 2 min at 94 °C followed by 30 cycles of 94 °C for 1 min, 55 °C for 1 min and 72 °C for 2 min. This was followed by a final extension step at 72 °C for 7 min. Resulting PCR products were sent to the GenePool sequencing facility (University of Edinburgh) for full sequencing on an ABI3730 (Applied Biosystems), using the M13F primer. Sequences were aligned by ClustalW in MEGA 4 (Molecular Evolutionary Genetics Analysis; Tamura *et al.*, 2007) and base pairs checked manually using BioEdit (http://www.mbio.ncsu.edu/BioEdit/bioedit.html). Sequences were then subjected to nBLAST analysis (Altschul *et al*., 1990) using the National Center for Biotechnology nucleotide (NCBI nt) database (www.ncbi.nlm.nih.gov/) to identify most closely related sequences and to infer taxonomic identity.

Cloned inserts were then re-amplified by PCR, prior to purification using the QIAquick PCR purification kit (Qiagen, Crawley, UK), visualisation by gel electrophoresis to ensure the correct size fragment was amplified and DNA quantification using the Quant-It PicoGreen dsDNA quantitation assay, according to the manufacturer’s instructions (Invitrogen, Paisley, UK). Standard curves were then constructed, via serial dilution, ranging from 10^3^ to 10^7^ copies per microlitre for 16S rRNA, *nirS* and archaeal *amoA* genes, and from 10^2^ to 10^6^ copies per microlitre for anammox 16S rRNA and bacterial *amoA* genes. For each primer pair, q-PCR assays were carried out within a single assay plate, allowing direct comparison of absolute numbers between environmental DNA samples (Smith *et al.*, 2006). Each assay also contained triplicate no-template controls (NTC). q-PCR amplification mixtures contained 1 µl of template DNA, 12.5 µl of SYBR Green Quanti Fast master mix (Qiagen) and primers at final concentrations as shown in Table S2 and were made up to a total reaction volume of 25 µl with sterile water. q-PCR amplification and detection for all assays was carried out using an ABI 7000 sequence detection system (Applied Biosystems, Carlsbad CA, USA) with an initial denaturation for 5 mins at 95 °C, followed by 40 cycles of 95 ^o^C for 15 s and annealing temperatures as listed in Table S2 for 1 min. Gene copy numbers were quantified via comparison to standard curves using the ABI Prism 7000 sequence detection software (see Table S2). For each primer set, q-PCR assays were optimised, and specificity of the q-PCR for the production of a single product was confirmed by performing melting curve analysis at the end of the 40 cycles (Ririe *et al.*, 1997). Gel electrophoresis of the q-PCR products further confirmed that only one product of the correct size was amplified. Sequence analysis of clone libraries generated using each set of primers detected only sequences related to the specific target gene of interest, confirming the specificity of each q-PCR assay for each target gene.

Observed gene abundance data are summarised in the Table S3, and full data are available from the Dryad Digital Repository (doi: 10.5061/dryad.k291r).

*Environmental data*

Environmental data (Fig. S1) were collected by Plymouth Marine Laboratory as part of the NERC National Capability funded Western Channel Observatory, and are available in full from the British Oceanographic Data Centre (BODC) (Woodward *et al*., 2013).

### *Statistical analyses*

All statistical analyses were performed in PRIMER 6.1 (Clarke & Gorley, 2006) or PRIMER 6.1 using the PERMANOVA+ add-on (beta version, Anderson *et al.*, 2008), on Bray-Curtis similarity matrices calculated from log(x+1)-transformed data. The RELATE test was used to compare gene abundance resemblance matrices with model matrices whose cells were populated according to the “distance” between samples. For model matrices, sample months were labelled 1 (July 2009) to 7 (July 2010), and the distance between samples was then calculated either in a linear series from 1 to 7 (test of seriation), or in a cyclical pattern so that 1 and 7 represent overlapping points (test of cyclicity).

Table S1: Sediment sampling strategy.

| **Label** | **Sampling date** | **Sediment location** | **No. replicates** |
| --- | --- | --- | --- |
| Jul-09 | 21.07.2009 | Surface  Burrow | 4  5 |
| Sep-09 | 29.09.2009 | Surface  Burrow | 5  5 |
| Nov-09 | 10.11.2009 | Surface  Burrow | 5  5 |
| Jan-10 | 11.01.2010 | Surface  Burrow | 5  5 |
| Mar-10 | 04.03.2010 | Surface  Burrow | 5  5 |
| May-10 | 27.05.2010 | Surface  Burrow | 5  5 |
| Jul-10 | 27.07.2010 | Surface  Burrow | 5  4 |

**Table S2:** Primer pairs and reaction conditions used for quantitative polymerase chain reaction (q-PCR) assays. q-PCR amplification and detection for all assays was carried out using an ABI 7000 sequence detection system (Applied Biosystems, Carlsbad CA, USA) with an initial denaturation for 5 mins at 95 °C, followed by 40 cycles of 95 °C for 15 s and annealing temperatures as listed below for 1 min. All reactions were carried out in 25 µl final volume; final primer concentrations are shown for this reaction volume. For each reaction, the standard curve was calculated using the ABI Prism 7000 sequence detection software. From the standard curve, the slope (*m)*, *y* intercept and coefficient of determination (*r^2^*) recorded and used to calculate the efficiency of the amplification (E) using the equation E = (10^1/^*^m^* -1)*100. Values for calculating the efficiency of each reaction are given, as well as the threshold cycle value (*C_T_*), which was determined using automatic analysis settings.

| **Target gene** | **Primer pair** | **Reference** | **Primer sequence,**  **5’ - 3’** | **Final conc., nM** | **Annealing temp., ^o^C** | **Fragment size, bp** | ***r^2^*** | ***y*** | **E,**  **%** | ***C_T_*** |
| --- | --- | --- | --- | --- | --- | --- | --- | --- | --- | --- |
| Bacterial 16S rRNA | Bact 1369F  Prok 1492R  (“BACT2”) | Suzuki *et al.*, 2000 | cggtgaatacgttcycgg  ggwtaccttgttacgactt | 300  900 | 60.0 | 123 | 0.998 | 37.90 | 92.0 | 25.3 |
| Archaea 16S rRNA | Parch519f  ARC915r | Coolen *et al.*, 2004 | cagccgccgcggtaa  gtgctcccccgccaattcct | 300  - | 63.0 | 396 | 0.999 | 37.27 | 88.5 | 33.5 |
| betaproteobacterial *amoA* | amoA1F-new  amoA2R-new | Stephen *et al.*, 1996  Hornek *et al.*, 2006 | GGGGHTTYTACTGGTGGT  CCCCTCBGSAAAVCCTTCTTC | 900  - | 61.5 | 490 | 0.998 | 38.75 | 83.6 | 37.7 |
| archaeal *amoA* | Arch-amoA-for  Arch-amoA-rev | Wuchter *et al*., 2006 | CTGAYTGGGCYTGGACATC  TTCTTCTTTGTTGCCCAGTA | 300  - | 58.5 | 256 | 0.994 | 42.20 | 77.2 | 36.6 |
| bacterial *nirS* | nirS1F  nirS3R | Braker *et al.*, 1998 | CCTAYTGGCCGCCRCART  GCCGCCGTCRTGVAGGAA | 900  - | 62.0 | 256 | 0.999 | 35.94 | 95.7 | 36.6 |
| anammox 16S rRNA | AMX 368F  AMX 820R | Jayakumar *et al.*, 2009 | Ttcgcaatgcccgaaagg  AAAYCCCTCTACTTAGTGCCC | 900  - | 59.0 | 452 | 0.999 | 35.92 | 93.5 | 33.2 |

**Table S3:** Seasonal variation in the average gene abundance (gene copies g^-1^ wet sediment) in burrow (B) and surface (S) sediments, and comparison to abundances reported in the literature for similar marine environments and using similar gene primers. Full data are available from the Dryad Digital Repository (doi: 10.5061/dryad.k291r). Absolute gene abundances were calculated from standard curves using the *r^2^*, *y* intercept and efficiency values given in Table S2.

|  |  | **Jul-09** | **Sep-09** | **Nov-09** | **Jan-10** | **Mar-10** | **May-10** | **Jul-10** | **Range** | **Previously reported range** | **Reference** |
| --- | --- | --- | --- | --- | --- | --- | --- | --- | --- | --- | --- |
| **Bacterial 16S rRNA** | **B** | 2.38 x 10^8^ | 2.89 x 10^8^ | 4.02 x 10^8^ | 4.95 x 10^8^ | 3.93 x 10^8^ | 3.40 x 10^8^ | 2.06 x 10^8^ | 10^7^ to 10^8^ | 10^8^ to 10^11^ | Schippers & Neretin, 2006; Leloup *et al*., 2007; Dang *et al*., 2010 |
|  | **S** | 3.24 x 10^8^ | 3.11 x 10^8^ | 3.01 x 10^8^ | 2.66 x 10^8^ | 6.82 x 10^8^ | 2.41 x 10^8^ | 2.78 x 10^8^ | 10^7^ to 10^9^ |  |  |
| **Archaeal 16S rRNA** | **B** | 7.63 x 10^6^ | 2.06 x 10^7^ | 2.37 x 10^7^ | 4.45 x 10^7^ | 3.61 x 10^7^ | 2.22 x 10^7^ | 8.80 x 10^6^ | 10^6^ to 10^7^ | 10^6^ to 10^7^ | Borrel *et al*., 2012 |
|  | **S** | 2.77 x 10^7^ | 1.38 x 10^7^ | 1.28 x 10^7^ | 1.08 x 10^7^ | 2.67 x 10^7^ | 6.18 x 10^6^ | 2.43 x 10^7^ | 10^6^ to 10^7^ |  |  |
| **Bacterial *amoA*** | **B** | 9.22 x 10^5^ | 1.64 x 10^7^ | 3.66 x 10^6^ | 9.33 x 10^6^ | 1.91 x 10^7^ | 1.09 x 10^7^ | 1.05 x 10^6^ | 10^5^ to 10^7^ | 10^5^ to 10^8^ | Beman *et al*., 2012, and references therein |
|  | **S** | 8.30 x 10^6^ | 2.67 x 10^6^ | 1.92 x 10^6^ | 2.20 x 10^6^ | 9.81 x 10^6^ | 1.66 x 10^6^ | 1.86 x 10^7^ | 10^5^ to 10^7^ |  |  |
| **Archaeal *amoA*** | **B** | 6.17 x 10^6^ | 9.51 x 10^6^ | 1.25 x 10^7^ | 3.10 x 10^7^ | 5.59 x 10^7^ | 3.91 x 10^7^ | 8.49 x 10^6^ | 10^6^ to 10^8^ | 10^4^ to 10^7^ | Beman *et al*., 2012, and references therein |
|  | **S** | 1.60 x 10^7^ | 8.42 x 10^6^ | 6.63 x 10^6^ | 1.78 x 10^7^ | 4.84 x 10^7^ | 7.95 x 10^6^ | 4.76 x 10^7^ | 10^6^ to 10^7^ |  |  |
| ***nirS*** | **B** | 1.41 x 10^7^ | 2.25 x 10^7^ | 1.66 x 10^7^ | 2.92 x 10^7^ | 3.39 x 10^7^ | 1.87 x 10^7^ | 9.36 x 10^6^ | 10^6^ to 10^7^ | 10^6^ to 10^8^ | Abell *et al*., 2010; Mosier & Francis, 2010 |
|  | **S** | 2.02 x 10^7^ | 1.95 x 10^7^ | 1.61 x 10^7^ | 1.47 x 10^7^ | 3.79 x 10^7^ | 1.31 x 10^7^ | 2.29 x 10^7^ | 10^6^ to 10^7^ |  |  |
| **anammox 16S rRNA** | **B** | 2.65 x 10^4^ | 2.55 x 10^5^ | 1.46 x 10^5^ | 3.55 x 10^5^ | 8.75 x 10^5^ | 6.74 x 10^5^ | 3.20 x 10^4^ | 10^4^ to 10^6^ | 10^5^ to 10^8^ | Dalsgaard *et al*., 2003; Schmid *et al*., 2007; Dang *et al*., 2010 |
|  | **S** | 1.45 x 10^5^ | 6.76 x 10^4^ | 4.21 x 10^4^ | 4.84 x 10^4^ | 1.36 x 10^5^ | 2.54 x 10^4^ | 1.30 x 10^6^ | 10^4^ to 10^6^ |  |  |


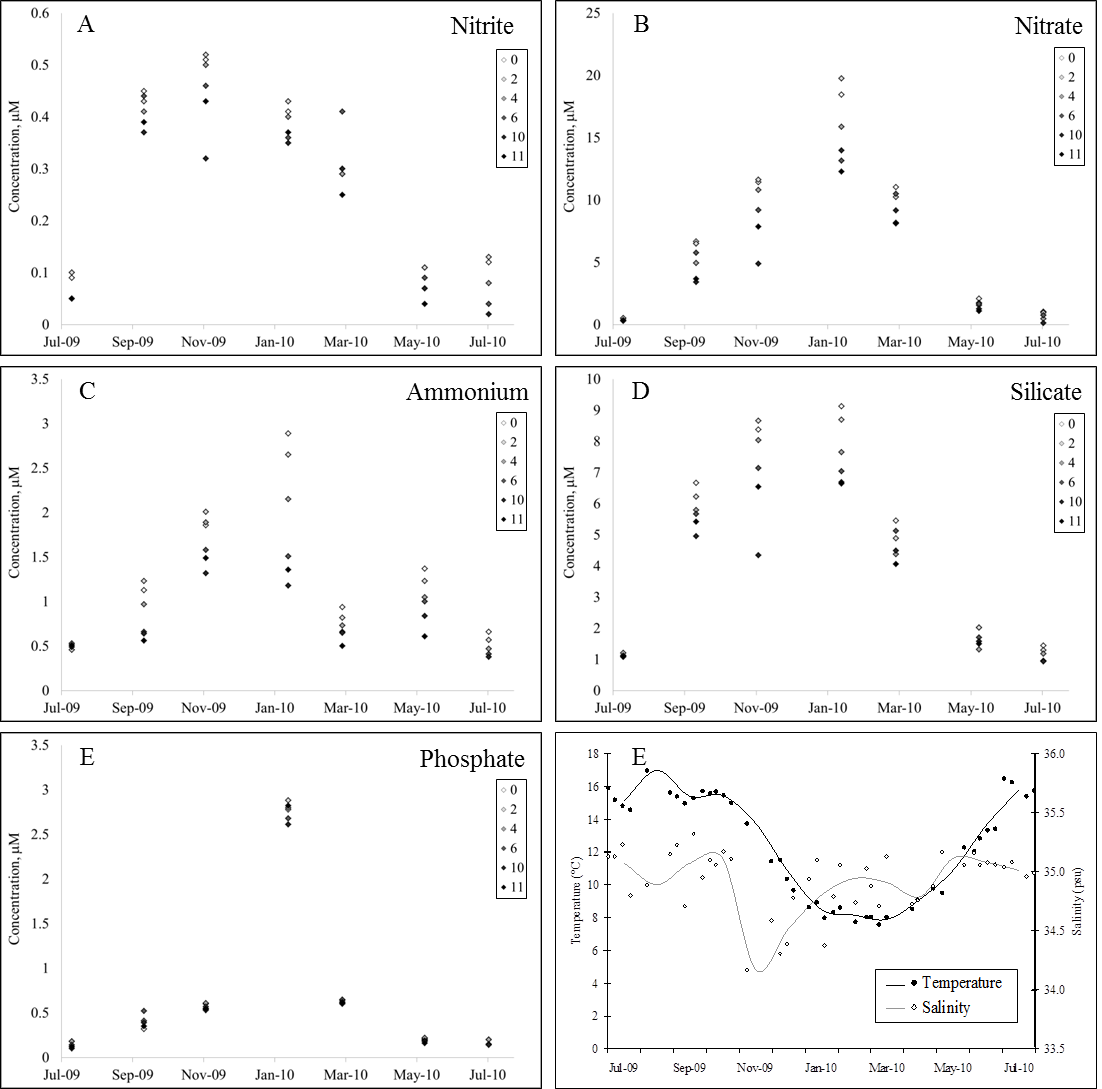


Figure S1: Variation in monthly pelagic nutrient concentrations in Jennycliff Bay (5.3497 N, 04.1331 W) in the Western English Channel. (A) nitrite, (B) nitrate, (C) ammonium, (D) silicate, (E) phosphate. Inset legends show water depth in metres. Data are from the PML Benthic Survey Data Inventory (Woodward *et al*., 2013). (F) Variation in monthly temperature (closed circles; black line) and salinity (open diamonds; grey line) for the L4 site (50.225 N, 04.1944 W), in the Western English Channel, with lines connecting the average value for each month. Data are from the Western Channel Observatory Data Inventory (Fishwick, 2013).

**References**

Abell GC, Revill AT, Smith C, Bissett AP, Volkman JK, Robert SS. (2010). Archaeal ammonia oxidizers and *nirS*-type denitrifiers dominate sediment nitrifying and denitrifying populations in a subtropical macrotidal estuary. ISME J 4: 286-300.

Altschul S, Gish W, Miller W, Myers E, Lipman D. (1990). Basic local alignment search tool. J Mol Biol 215: 403-410.

Anderson M, Gorley R, Clarke K. (2008). PERMANOVA+ for PRIMER: guide to software and statistical methods. PRIMER-E, Plymouth.

Beman MJ, Bertics VJ, Braunschweiler T, Wilson JM. (2012). Quantification of ammonia oxidation rates and the distribution of ammonia-oxidising Archaea and Bacteria in marine sediment depth profiles from Catalina Island, California. Front Microbiol 3: 263.

Borrel G, Lehours A-C, Crouzet O, Jézéquel D, Rockne K, Kulczak A, *et al.* (2012). Stratification of archaea in the deep sediments of a freshwater meromictic lake: Vertical shift from methanogenic to uncultured archaeal lineages. PLoS ONE 7: e43346.

Braker G, Fesefeldt A, Witzel K-P. (1998). Development of PCR primer systems for amplification of nitrite reductase genes (*nirK* and *nirS*) to detect denitrifying bacteria in environmental samples. Appl Environ Microbiol 64: 3769-3775.

Clarke KR, Gorley RN. (2006). PRIMER v6: User Manual/Tutorial, Vol. PRIMER-E, Plymouth.

Coolen MJL, Hopmans EC, Rijpstra WIC, Muyzer G, Schouten S, Volkman JK, *et al.* (2004). Evolution of the methane cycle in Ace Lake (Antarctica) during the Holocene: response of methanogens and methanotrophs to environmental change. Org Geochem 35: 1151-1167.

Dalsgaard T, Canfield DE, Petersen J, Thamdrup B, Acuna-Gonzalez J. (2003). N_2_ production by the anammox reaction in the anoxic water column of Golfo Dulce, Costa Rica. Nature 422: 606–608.

Dang H, Chen R, Wang L, Guo L, Chen P, Tang Z, *et al.* (2010). Environmental factors shape sediment anammox bacterial communities in hypernutrified Jiaozhou Bay, China. Appl Environ Microbiol 76: 7036-7047.

Fishwick JR. (2013). CTD profiles (depth, pressure, temperature, salinity, potential temperature, density, fluorescence, transmissance, downwelling PAR, dissolved oxygen concentration) binned to 0.5 m and 0.25 m at sites L4 and E1 in the Western English Channel between January 2002 and December 2012. British Oceanographic Data Centre - Natural Environment Research Council UK.

Hornek R, Pommerening-Röser A, Koops H-P, Farnleitner AH, Kreuzinger N, Kirschner A, *et al.* (2006). Primers containing universal bases reduce multiple *amoA* gene specific DGGE band patterns when analysing the diversity of beta-ammonia oxidizers in the environment. J Microbiol Meth 66: 147-155.

Jayakumar A, Naqvi SWA, Ward BB. (2009). Distribution and relative quantification of key genes involved in fixed nitrogen loss from the Arabian Sea oxygen minimum zone. Geophys Monogr 185: 187-203.

Laverock B, Smith CJ, Tait K, Osborn AM, Widdicombe S, Gilbert JA. (2010). Bioturbating shrimp alter the structure and diversity of bacterial communities in coastal marine sediments. ISME J 4: 1531–1544.

Leloup J, Loy A, Knab NJ, Borowski C, Wagner M, Jørgensen BB. (2007). Diversity and abundance of sulfate-reducing microorganisms in the sulfate and methane zones of a marine sediment, Black Sea. Environ Microbiol 9: 131-142.

Mosier AC, Francis CA. (2010). Denitrifier abundance and activity across the San Francisco Bay estuary. Environ Microbiol Reports 2: 667-676.

Ririe KM, Rasmussen RP, Wittwer CT. (1997). Product differentiation by analysis of DNA melting curves during the polymerase chain reaction. Anal Biochem 245: 154-160.

Schippers A, Neretin LN. (2006). Quantification of microbial communities in near-surface and deeply buried marine sediments on the Peru continental margin using real-time PCR. Environ Micriobiol 8: 1251-1260.

Schmid MC, Risgaard-Petersen N, van de Vossenberg J, Kuypers MMM, Lavik G, Petersen J, *et al.* (2007). Anaerobic ammonium-oxidising bacteria in marine environments: widespread occurrence but low diversity. Environ Microbiol 9: 1476-1484.

Smith CJ, Nedwell DB, Dong LF, Osborn AM. (2006). Evaluation of quantitative polymerase chain reaction-based approaches for determining gene copy and gene transcript numbers in environmental samples. Environ Microbiol 8: 804-815.

Stephen JR, McCaig AE, Smith Z, Prosser JI, Embley TM. (1996). Molecular diversity of soil and marine 16S rRNA gene sequences related to beta-subgroup ammonia-oxidizing bacteria. Appl Environ Microbiol 62: 4147-4154.

Suzuki MT, Taylor LT, DeLong EF. (2000). Quantitative analysis of small-subunit rRNA genes in mixed microbial populations via 5'-nuclease assays. Appl Environ Microbiol 66: 4605-4614.

Tamura K, Dudley J, Nei M, Kumar S. (2007). MEGA4: Molecular Evolutionary Genetics Analysis (MEGA) software version 4.0. Mol Biol Evol 24: 1596–1599.

Woodward EMS, Harris C, Tait K. (2013). PML Benthic Survey water column nutrient concentrations from 4 different benthic study sites off Plymouth, South West England, between 2008 and 2011. British Oceanographic Data Centre - Natural Environment Research Council UK. doi:10.5285/ed97f20f-8d7e-11e2-b183-1803734a77fb

Wuchter C, Abbas B, Coolen MJL, Herfort L, Bleijswijk Jv, Timmers P, *et al.* (2006). Archaeal nitrification in the ocean. Proc Natl Acad Sci USA 103: 12317-12322.
